# 小细胞肺癌合并副肿瘤性边缘叶脑炎病例分析

**DOI:** 10.3779/j.issn.1009-3419.2019.03.02

**Published:** 2019-03-20

**Authors:** 明一 邸, 力 张

**Affiliations:** 100730 北京，北京协和医院呼吸内科 Department of Respiratory Medicine, Peking Union Medical College Hospital, Beijing 100730, China

**Keywords:** 肺肿瘤, 副肿瘤性边缘叶脑炎, 副肿瘤综合征, Lung neoplasms, Paraneoplastic limbic encephalitis, Paraneoplastie syndmme

## Abstract

**背景与目的:**

本研究旨在探讨小细胞肺癌（small cell lung cancer, SCLC）合并副肿瘤性边缘叶脑炎（paraneoplastic limbic encephalitis, PLE）的诊断及治疗要点。

**方法:**

收集北京协和医院1980年1月-2017年5月收治的15例SCLC合并PLE患者的、临床资料，并分析其症状、实验室检查数据并随访其预后。

**结果:**

PLE是较为罕见的疾病类型，在SCLC中的发病率约为0.842%，该数据可能因误诊、漏诊而被低估；中年吸烟男性患者为高发人群，肿瘤-淋巴结-转移（tumor-node-metastasis, TNM）分期多偏晚；神经系统症状多为典型的边缘叶脑炎症状，包括不同程度的短期记忆力丧失、癫痫发作及不同程度的精神异常；神经系统症状多先于肿瘤发现或呼吸系统症状出现，发病到确诊中位时间约2个月；实验室检查中多有血清抗体（抗Hu、GABA-R-Ab）、脑脊液、头磁共振成像（magnetic resonance imaging, MRI）及脑电图异常；影像学尤其是计算机断层扫描（computed tomography, CT）是筛查原发肿瘤较好的手段，病理确诊主要依靠支气管镜；针对原发肿瘤的治疗比免疫治疗能够更有效地缓解神经系统症状。

**结论:**

副肿瘤性边缘叶脑炎是一种较为罕见的由恶性肿瘤导致的远隔性神经系统副肿瘤综合征，常以边缘神经系统症状为特征性表现，多数与肺癌（尤其是SCLC）相关，其神经系统异常多早于肿瘤诊断，早期诊断及针对原发肿瘤的治疗将提高获益。

## Abstract (en)

**Background and objective:**

The aim of this study was to investigate the clinical features of paraneoplastic limbic encephalitis (PLE) with small cell lung cancer (SCLC) and improve clinical diagnosis and treatment.

**Methods:**

The clinical data of 15 patients with SCLC combined with PLE from January 1980 to May 2017 were collected from Beijing Union Hospital. Their symptoms and laboratory data were analyzed and the prognosis of the patients was followed.

**Results:**

PLE is a rare disease, the incidence rate in SCLC is about 0.842%. The data may be underestimated because of misdiagnose or missed diagnosis; High incidence crowd of the disease is the middle-aged male smoker, the tumor-node-metastasis (TNM) stages of them are later than others; Typical neurological symptoms include varying degrees of short-term memory loss, seizures and varying degrees of mental disorders; neurological symptoms usually occur before the onset of cancer or respiratory symptoms appear, an average of about 2 months be taken from onset to diagnosis; Serum antibody (anti-Hu, GABA-R-Ab), cerebrospinal fluid, head magnetic resonance imaging (MRI) and electroencephalogram (EEG) of the patients has abnormalities; Videography, especially computed tomography (CT) is a good means of screening the primary tumor, pathology diagnosis mainly rely on bronchoscopy; The treatment of primary tumors can be more effective in alleviating the nervous system symptoms than immunotherapy.

**Conclusion:**

Paraneoplastic limbic encephalitis is a rare paraneoplastic syndrome in nervous system caused by malignant neoplasms often characterized by facial neurological symptoms. The disease are usually associated with lung cancer (especially SCLC). Its nervous system symptoms occur earlier than the tumor diagnosis. Early diagnosis and treatment for primary tumors will increase the benefit.

副肿瘤综合征（paraneoplastie syndmme, PNS）是由于恶性肿瘤造成的远隔表现，与肿瘤自身的侵犯与转移无关，目前认为其发生可能与免疫介导机制相关^[1]^。副肿瘤性边缘叶脑炎（paraneoplastic limbie encephalitis, PLE）是一种罕见的由恶性肿瘤导致的远隔性神经系统PNS，与肿瘤转移、代谢紊乱、营养缺乏、感染、凝血障碍、肿瘤治疗毒副作用等无关，其发生可能与抗神经元抗体相关源T淋巴细胞介导为主的一种自身免疫反应有关；选择性地影响边缘系统结构，包括海马、下丘脑和杏仁核，常表现为急性或亚急性加重的神经功能损害^[2]^；PLE的发生多数与肺癌，尤其是小细胞肺癌（small cell lung cancer, SCLC）相关^[3]^。由于发生多早于肿瘤确诊，容易被误诊。现总结北京协和医院1980年1月-2017年5月收治的15例小细胞肺癌合并边缘叶脑炎的患者的临床资料，分析其临床特征、诊疗要点并随访其预后，为后续临床诊治提供参考。

## 1 对象与方法

### 1.1 对象

回顾性分析1980年1月-2017年5月我院收治的SCLC患者1, 781例，根据2004年神经系统PNS欧洲工作网制定的PLE诊断标准，从中筛选出PLE患者共15例。所有患者均经活检病理确诊SCLC。其中男性14例，女性1例；年龄41岁-79岁，中位年龄56岁。

### 1.2 方法

收集所有患者的临床资料，回顾性总结并分析本组患者的人口学特征、原发病及神经系统症状、实验室检查结果、治疗及部分预后特征。

### 1.3 统计学分析

采用统计学软件SPSS 21.0进行数据分析。计数资料采用率（%）表示。

## 2 结果

### 2.1 一般资料

本次所收录的15例SCLC合并边缘叶脑炎患者的诊断结合了病史、临床症状、辅助检查等综合判断。15例患者中，根据患者的首发症状，初诊科室为神经内科5例、呼吸科5例及急诊科2例（分别以咯血和低钠血症为首发症状），胸外科2例，内分泌科1例。发病到确诊的中位时间约2个月（65 d）；8例在首诊时即疑诊为PLE。入组患者中位年龄56（41-79）岁，其中男性14例，女性仅1例；多有长期大量吸烟史；肿瘤分期方面：SCLC局限期13例，广泛期仅2例。

### 2.2 首发症状及临床表现

15例患者中仅2例以呼吸系统症状为首发症状（咳嗽1例，发现肺部结节增大1例），11例以神经系统症状为首发症状，2例以低钠血症起病；其中5例直至确诊时仍无呼吸系统症状。

患者的神经系统症状为典型的边缘叶脑炎症状，包括不同程度的短期记忆力丧失13例（86.67%），癫痫发作11例（73.33%），不同程度的精神异常7例（包括心境行为改变、运动障碍、认知障碍等，46.67%）；15例患者中11例均合并抗利尿激素分泌不当综合征（syndrome of inappropriate secretion of antidiuretic hormone, SIADH）。首诊时血钠波动于101 mmol/L-120 mmol/L。患者神经系统异常呈急性或亚急性进行性加重。呼吸系统症状主要为咳嗽（10例）、咯血（2例）、呼吸困难（1例）、肺部结节增大（1例）等非特异性症状。

### 2.3 实验室检查

15例患者同时接受了脑脊液、脑电图、头颅磁共振成像（magnetic resonance imaging, MRI）等检查，结果提示符合PLE改变，并除外了脑转移瘤、代谢性脑病、放射性脑炎、脑血管病、颅内感染等情况。其中脑脊液异常13例（86.67%），脑电图异常7例（46.67%），头颅MRI提示单侧或双侧颞叶非肿瘤性异常信号8例（53.33%）。肺癌的发现依照影像学[胸部X线片/计算机断层扫描（computed tomography, CT）或正电子发射计算机断层显像（positron emission tomography/CT, PET/CT）]，确诊主要依靠纤维支气管镜下经支气管肺活检8例，另有锁骨上淋巴结活检术确诊1例，肺叶切除术确诊2例。其中4例根据PET/CT、肿瘤指标等疑诊SCLC，无明确病理证据。分期方面：SCLC的肿瘤分期依靠手术病理、胸部CT以及PET/CT，15例患者诊断时肿瘤分期均为IIIa期或更晚期。15例患者中5例患者压力增高，其余正常；脑脊液性状均为无色透明；蛋白正常2例，轻度增高7例；脑脊液抗-Hu（+）6例，其中4例同时均有血抗Hu阳性。3例患者脑脊液GABA-R-Ab（+），同时血GABA-R-Ab（+）。本研究收录的15例患者中4例血抗Hu阳性，5例患者血GABA-R-Ab（+）。多数抗体在血清与脑脊液中同时存在。影像学检查方面：头颅MRI提示非肿瘤性异常信号12例，其中8例集中位于双侧或单侧颞叶。其他位于侧脑室旁、双侧额叶、中央区等部位，仅3例患者头MRI无明显异常表现。15例患者中出现脑电图（electroencephalogram, EEG）异常7例，包括背景慢波化，尖波、尖慢复合波局限性出现，局部α节律消失，θ波功率增高等，上述表现均不特异，且所有病例均未见异常痫性电活动。实验室检查结果汇总见表 1。

**1:** 小细胞肺癌合并边缘叶脑炎患者抗体、脑脊液、脑电图及影像学检查结果 Antibodies, CSF analyses, EEGs, and imaging results in small cell lung cancer (SCLC) patients with paraneoplastic limbie encephalitis (PLE)

Examinations		Positive	Negative	Not available
Antibodies in serum	Anti-GABA-R	5	1	9
	Anti-Hu	4	3	8
	Anti-Yo	1	6	8
Cerebro-spinal fuid (CSF)	Anti-GABA-R	6	3	6
	Anti-Hu	6	3	6
	CSF pressure	5	4	6
	CSF protein	7	2	6
	Increased number of CSF cells number	4	5	6
Electroencephalograph (EEG)		7	2	6
Magnetic resonance imaging (MRI)		12	3	0

### 2.4 治疗及预后

15例患者均不同疗程地接受抗癫痫药物治疗。其中10例接受类固醇激素治疗（其中3例接受甲强龙激素冲击治疗）；3例接受静脉注射免疫球蛋白（intravenous immunoglobulin, IVIG）治疗（均同时或序贯接受了口服激素治疗）。14例患者在确诊SCLC后接受一线依托泊苷+铂二联方案化疗（其中6例联合放疗），1例患者未接受任何治疗。化疗后患者神经系统症状均获得不同程度的缓解（其中神经系统症状完全缓解7例，部分缓解5例）。从使用抗癌药物到开始出现神经系统症状缓解的中位反应时间为25 d。14例接受治疗的患者中7例神经系统症状完全缓解（complete response, CR）。肿瘤治疗及疗效方面：在接受抗肿瘤治疗的14例患者中，12例在一线治疗后可评估肺癌的疗效，总有效率为80%。2例CR，7例部分缓解（partial response, PR），3例稳定（stable disease, SD）。中位随访时间为20.2（1.5-57）个月。见图 1。

**1:**
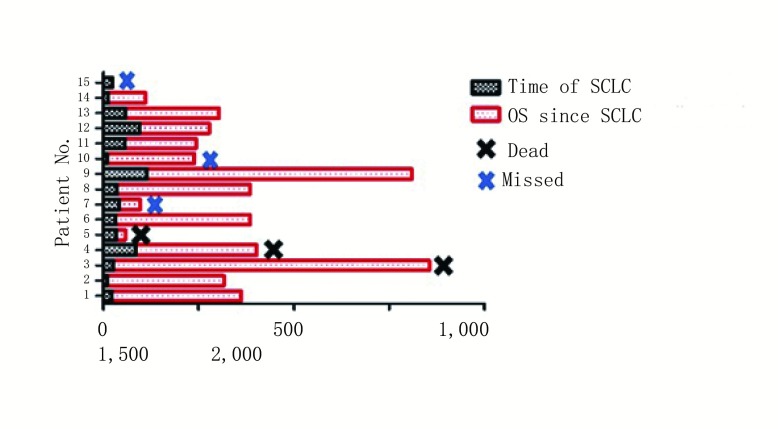
患者生存情况随访 Survival of PLE patients

## 3 讨论

PLE为罕见病变，发病率未见报道，本研究结果显示PLE在SCLC中发生率为0.842%，由于可能存在漏诊及误诊，该数据可能被低估。根据本组资料，中年男性吸烟患者为其高发人群，肿瘤分期多为局部晚期以上。PLE以急性或亚急性心境和行为改变、短期记忆问题、复杂-部分性癫痫发作和认知功能障碍为特点^[4]^，也可能发生下丘脑功能障碍，表现为过热、嗜睡和内分泌异常。约2/3的边缘叶脑炎患者发生神经系统多灶性受累（如脑脊髓炎）。PLE的诊断依据欧洲工作网制定的诊断标准：①急性或亚急性（数天或最长达12周）起病的癫痫发作、短时记忆丧失、意识混乱和精神症状；②边缘系统受累的神经病理学证据或影像学证据；③除外其他病因所致的边缘叶功能障碍，包括肿瘤脑转移、代谢性脑病、放射性脑炎、脑血管病、颅内感染、凝血障碍、化疗不良反应等；④出现神经系统症状5年内证实肿瘤的诊断或出现边缘叶功能障碍的典型症状时伴极具特征性的肿瘤抗体阳性^[5]^。与PLE有关的最常见的肿瘤是肺癌（尤以SCLC多见），其他如精原细胞瘤、睾丸肿瘤、胸腺瘤、乳腺癌和霍奇金淋巴瘤亦可引起。神经系统症状通常在肿瘤发现的数周或数月之前出现。神经系统PNS的诊断从策略上可以归纳为两步：首先确定边缘叶脑炎，然后寻找肿瘤证据并除外其他病因。对于具有典型边缘叶脑炎症状的患者，尤其是中年吸烟男性，无论是否合并呼吸道症状都应考虑肺癌的可能，应积极寻找肿瘤证据。

SCLC相关的边缘叶脑炎特征性血清副肿瘤抗体包括Anti-Hu（ANNA-1）、Anti-Ri（ANNA-2）、Anti-CV2/CRMP5，部分特征性副肿瘤抗体包括Anti-Zic 4和Anti-PCA2^[4]^。大多数SCLC患者的血清和脑脊液中有抗-Hu抗体，这些抗体阳性的患者出现副肿瘤性脑脊髓炎其他表现的比例更高^[5, 6]^。有研究^[15]^报道了73例抗Hu抗体阳性的PNS患者，其临床类型包括感觉神经病（55%）、小脑变性（22%）、边缘性脑炎（15%）和脑干脑炎（16%），23%伴有自主神经功能障碍如胃肠动力障碍（14%）；85%的患者发现肿瘤，其中77%为肺癌。根据既往的报道，抗Hu抗体检测对诊断PNS及其相关肿瘤具有较高的特异性（95%-100%）和敏感性（80%以上）^[16]^。本研究收录的病例中，血清抗-Hu阳性率为26.6%，阳性率较低，可能的原因是样本量过小，需待更大规模的临床数据以证实抗Hu抗体对PLE的预测价值。PLE的脑脊液检查包括细胞计数、蛋白和葡萄糖检测、病原学、细胞学等筛查。副肿瘤性脑炎的脑脊液检查最常见蛋白轻度升高（< 100 mg/dL）；少数患者有轻度淋巴细胞增多和/或更显著的蛋白升高^[6]^。本研究中，7例患者的脑脊液均提示炎症改变（淋巴细胞增多、寡克隆条带、免疫球蛋白成分增高或蛋白含量增多而无可测量免疫球蛋白），15例患者的脑脊液细胞学检查均未发现恶性细胞。PLE患者脑电图可显示单侧或双侧颞叶慢波或快波。常见非特异性脑电图异常包括局灶性或泛发性缓慢、癫痫样活动和周期性单侧性癫痫样放电（periodic lateralized epileptiform discharges, PLEDS）^[6, 7]^。这种活动在颞叶区域最大^[6]^。脑MRI有助于排除脑血管事件或转移性疾病以及其他疾病。PLE的特征性MRI发现包括在受影响的大脑区域（即单或双侧颞叶和/或脑干）FLAIR或T2加权像上的高信号；有时皮质下区域和小脑也受到影响。但这些MRI表现对疾病的诊断无特异性^[6]^。PET在急性期有可显示内侧颞叶的高代谢^[8]^，较MRI更灵敏^[9, 10]^，同时还可在发现肿瘤、评估肿瘤方面起一定作用。

在确诊边缘叶脑炎后，患者应及时进行隐匿性恶性肿瘤筛查。大多数PLE潜在肿瘤为SCLC，因此应优先选择胸部影像学检查。影像学（胸部X线、胸部CT、PET/CT等）对原发肿瘤的发现均具有积极意义。纤维支气管镜检查是获取病理诊断的有效方法。本组15例中有8例通过纤维支气管镜检查明确病理诊断。针对原发肿瘤的抗肿瘤治疗能够有效缓解患者的神经系统症状，使神经系统功能部分或完全恢复。尽管目前尚无针对PLE患者的免疫治疗对照研究，对于正在接受肿瘤治疗的患者，免疫抑制治疗（糖皮质激素、IVIG和血浆置换）或可有效^[11]^。当其他免疫治疗无效时，环磷酰胺或利妥昔单抗可能有效^[13, 14]^。对于已除外感染，具有特征性临床和放射学表现的患者，其免疫抑制治疗的开始不必等待肿瘤诊断或抗体表征结果^[12]^。伴有癫痫发作的患者应使用抗癫痫药物（antiepileptic drugs, AED）控制症状，大多数患者在接受系统治疗后不需要长期AED治疗^[13]^。

PLE患者的总体预后高度可变，取决于原发肿瘤类型、分期及神经综合征的严重程度。延误诊断和治疗与较差预后有关。积极的抗肿瘤治疗比免疫抑制治疗能更有效地缓解神经系统症状，并能够改善预后。抗-Hu抗体阳性、神经系统症状缓解不完全以及肺癌分期偏晚三者之间具有相关性，均与预后较差相关^[3]^。
